# Are antidementia drugs associated with reduced mortality after a hospital emergency admission in the population with dementia aged 65 years and older?

**DOI:** 10.1016/j.trci.2019.07.011

**Published:** 2019-09-03

**Authors:** Simona Hapca, Jennifer Kirsty Burton, Vera Cvoro, Emma Reynish, Peter T. Donnan

**Affiliations:** a Population Health and Genomics, School of Medicine, University of Dundee, Dundee, UK; b Institute of Cardiovascular and Medical Sciences, Glasgow Royal Infirmary, University of Glasgow, Glasgow, UK; c NHS Fife, Kirkcaldy, UK; d Dementia and Ageing Research Group, Faculty of Social Science, University of Stirling, Stirling, UK; e Geriatric Medicine, NHS Lothian, Edinburgh, UK; f Population Health and Genomics, School of Medicine, University of Dundee, Dundee, UK

**Keywords:** Antidementia medication, Acetylcholinesterase inhibitors, Memantine, Emergency admission, Mortality

## Abstract

**Introduction:**

People with dementia experience poor outcomes after hospital admission, with mortality being particularly high. There is no cure for dementia; antidementia medications have been shown to improve cognition and function, but their effect on mortality in real-world settings is little known. This study examines associations between treatment with antidementia medication and mortality in older people with dementia after an emergency admission.

**Methods:**

The design is a retrospective cohort study of people aged ≥65 years, with a diagnosis of dementia and an emergency hospital admission between 01/01/2010 and 31/12/2016. Two classes of antidementia medication were considered: the acetylcholinesterase inhibitors and memantine. Mortality was examined using a Cox proportional hazards model with time-varying covariates for the prescribing of antidementia medication before or on admission and during one-year follow-up, adjusted for demographics, comorbidity, and community prescribing including anticholinergic burden. Propensity score analysis was examined for treatment selection bias.

**Results:**

There were 9142 patients with known dementia included in this study, of which 45.0% (n = 4110) received an antidementia medication before or on admission; 31.3% (n = 2864) were prescribed one of the acetylcholinesterase inhibitors, 8.7% (n = 798) memantine, and 4.9% (n = 448) both. 32.9% (n = 1352) of these patients died in the year after admission, compared to 42.7% (n = 2148) of those with no antidementia medication on admission. The Cox model showed a significant reduction in mortality in patients treated with acetylcholinesterase inhibitors (hazard ratio [HR] = 0.78, 95% CI 0.72–0.85) or memantine (HR = 0.75, 95% CI 0.66–0.86) or both (HR = 0.76, 95% CI 0.68–0.94). Sensitivity analysis by propensity score matching confirmed the associations between antidementia prescribing and reduced mortality.

**Discussion:**

Treatment with antidementia medication is associated with a reduction in risk of death in the year after an emergency hospital admission. Further research is required to determine if there is a causal relationship between treatment and mortality, and whether “symptomatic” therapy for dementia does have a disease-modifying effect.

## Highlights

• Mortality in older people admitted with dementia is very high after an emergency hospital admission with about 40% of them dying within one year from admission.
• Less than half of patients admitted with dementia are in receipt of antidementia treatment on admission.
• Risk of death is reduced in patients treated with antidementia medication at the time of acute admission, which is not explained by their age, sex, comorbidities, or other drug prescribing.

## 1 Introduction

With the aging population, the number of people living with dementia is forecasted to increase [1]. Dementia is known to shorten life expectancy [2], and there is currently no known cure or disease-modifying treatment. Symptomatic treatment is available for those with dementia due to Alzheimer's disease (AD). Two classes of medication are currently licensed: the acetylcholinesterase inhibitors (AChEIs: donepezil, galantamine, and rivastigmine) and the N-methyl-D-aspartate receptor antagonist, memantine [3], [4], [5]. The efficacy of these medications has been demonstrated in randomized controlled clinical trials, with most of them showing associations between antidementia drug use and improved cognition and functional ability [5], [6], [7], [8], [9], [10], [11], [12], [13]. In addition, some trials have shown prolonged independence and home living in people on antidementia medication compared to placebo [14], [15], [16]. Observational studies investigating long-term therapeutic effects of antidementia drugs in real-world settings have shown a reduced decline in cognition and function in those treated with antidementia medication [17], [18]. In 2011, NICE suggested that evidence of improvement in clinically meaningful outcomes for these medications was lacking [19]. In a recent systematic review and meta-analysis of randomized placebo-controlled clinical trials, the use of AChEIs was associated with reduced mortality [20], and the authors suggest this finding may indicate some disease-modifying effect of these drugs. This finding however is not consistent across all studies: some studies show a reduction in mortality in people who were prescribed antidementia medication [18], [21], [22], [23], one study shows a reduction in cardiovascular deaths [24], and another has failed to find an association [15].

It has been shown that mortality in people with dementia is not associated with disease severity, cognitive function, or functional ability [2], and we can therefore postulate that other factors in addition to disease progression may have a role. Most people with dementia die either in an acute hospital or care home [25]. Many people with dementia are admitted to care homes from the acute hospital [26]. In the acute hospital setting, people with dementia have poor outcomes; they have longer hospital stays [27], an increased risk of not returning home [26], and high mortality [28].

Population data from England show that around 20% of people with dementia die annually [29]. Those admitted to the acute hospital are at higher risk of death with the mortality rate in the year after admission approximately 40% [27], [30]. By examining the population with dementia who are at highest risk of death, we aim to target our study of the effects of medication on this enriched population. The aim is to examine the association between treatment with antidementia medication and mortality in this high-risk group, that is, older people with dementia after an emergency admission to the acute hospital.

## 2 Methods

### 2.1 Population and data

National population–based health care data sets were used to identify all residents of two Scottish Health Board regions (Tayside and Fife) with known dementia, aged 65 years and older, admitted to hospital as a medical emergency between 1/1/10 and 31/12/16. NHS Tayside and Fife provide care to a diverse rural and urban area with a population in 2017 of ∼775,000 which is approximately 14% of the population of Scotland [31]. The configuration of service provision for unscheduled emergency medical admissions of adults is via acute medical units with subsequent discharge or step down to appropriate medical wards after 12–24 hours.

An incident emergency admission cohort was selected to study those patients at the outset of their interaction with acute hospital services. This cohort was defined as those patients with a first emergency admission during the seven-year study period after the dementia diagnosis in those aged ≥65 on admission, with no previous emergency admission in the preceding year. Mortality was ascertained within the first year of follow-up from the date of the emergency admission.

People with dementia were identified based on the International Classification of Diseases (ICD-10) codes from Scottish Morbidity Records (SMR01 and SMR04), which are validated NHS Scotland routine data sets for general admissions and psychiatric admissions, respectively, and the community prescribing data. The latter data set was also used to identify people, who were in receipt of a licensed medication for dementia, and the duration of treatment. Two classes of antidementia medication were considered: AChEIs (donepezil, galantamine, and rivastigmine) and memantine.

Data for incident emergency admissions were identified from SMR01 data providing also admission and discharge dates and destinations (whether care home or private home) and discharge diagnosis (based on ICD-10 codes). Discharge diagnosis from all previous admissions was used to calculate each participant's comorbidities for case-mix adjustment. Thirteen comorbidities (excluding dementia) were considered for adjustment as described in the study by Quan et al. [32]; however, liver disease, paraplegia and hemiplegia, and AIDS were present in less than 1% of the incident cohort and so they were not included in the analysis.

Data on all community-dispensed prescriptions were used to create an additional multimorbidity score, calculated as the number of drugs (defined as the number of distinct British National Formulary subsections) prescribed to the patient 12 weeks before admission [33]. The community-dispensed prescribing data were also used to identify prescribed anticholinergic and sedative medication to calculate an anticholinergic burden (ACB) score for the same time period [34].

The Community Health Index data set (CHI—the NHS Scotland population based on General Practice registration) was used to ascertain mortality and to define participant age, sex, and postcode-defined socioeconomic status (measured using quintiles of the Scottish Index of Multiple Deprivation) on admission [35]. The CHI number (the NHS Scotland unique patient identifier) was used to deterministically link SMR01 to CHI, SMR04, and community-dispensed prescribing.

### 2.2 Primary outcome, treatment, and control groups

The primary outcome of the study was time to death within one-year follow-up from the incident emergency admission, with mortality being ascertained from patient date of death provided in the CHI records.

**Fig. 1:**
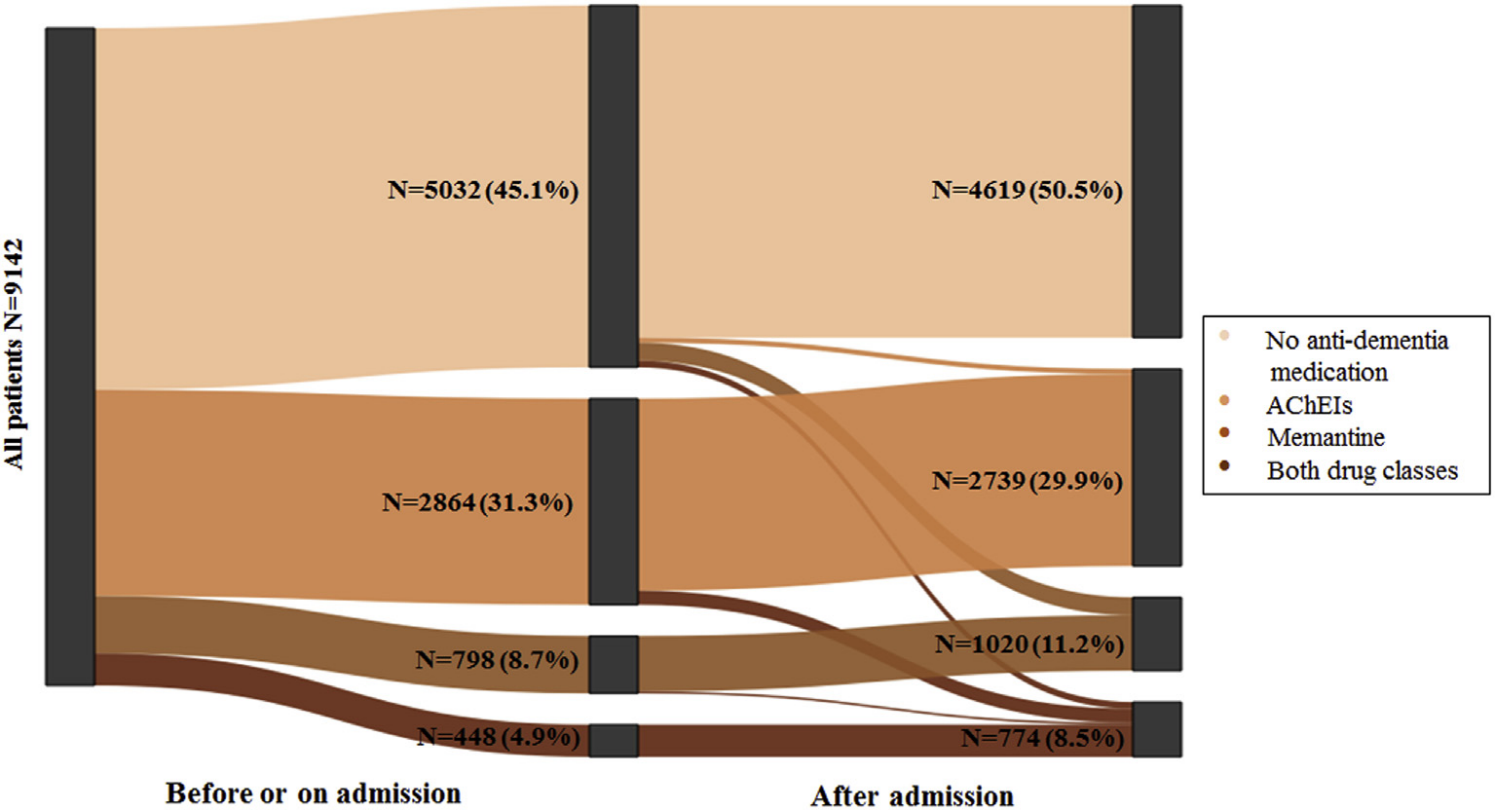
Antidementia prescribing rates before/on admission and after admission (analysis undertaken under the ITT principle). Abbreviations: AChEIs, acetylcholinesterase inhibitors; ITT, intention-to-treat.

To examine associations between antidementia drugs prescribing and outcomes in people admitted as medical emergencies, the treatment group was defined by intention-to-treat and included patients that were in receipt of antidementia medication before admission, on admission, or after admission. Specifically, the treatment group was divided into patients that were prescribed AChEIs only, memantine only, or both classes before, on or after admission (Fig. 1). The reference/control group included all remaining patients who were not even in receipt of an antidementia medication. Some patients in the treatment group were in receipt of antidementia medication before admission but this was not continued at the time of their admission or after admission; therefore, a secondary analysis was undertaken with the treatment group just including patients that were in receipt of antidementia medication at the time of their admission (defined as antidementia drug prescribed within the 12 weeks before admission) or after their admission.

### 2.3 Statistical analysis

Summary statistics based on proportions and their confidence intervals were used to estimate the distribution of patients that were in receipt of antidementia medication before or on admission (a binary variable indicating whether patients were prescribed either an AChEIs or memantine, or both before the incident emergency admission) according to their demographic characteristics, comorbidities, and polypharmacy. In addition, patients who were in receipt of antidementia medication on admission (defined as patients being prescribed an antidementia drug in the 12 weeks before admission) or started medication after admission were recorded separately. Associations between the rate of antidementia drug prescribing and patients' demographic characteristics, comorbidity, community prescribed drugs, and health board were examined with chi-square tests for association.

Associations between antidementia drug prescribing and time to mortality within one-year follow-up from admission were examined using a Cox proportional-hazards survival model. The Cox proportional-hazards model investigates the relationship of predictors and the time-to-event, in this case death. It assumes that the predictors have a multiplicative effect on the hazard and that this effect is constant over time. Also, owing to the regression framework of the model, hazard ratio estimates that are controlled for other covariates are possible. In this case, adjustment for demographics, comorbidity and community prescribing, ACB score, admission time, and type of emergency admission was undertaken. Assessment of the proportional-hazards assumption [36] showed that some Cox model covariates did not meet this assumption; therefore, for these covariates, piecewise constant time-varying coefficients were fitted to the data to estimate changes in hazard ratios over time [37].

The prescribing data showed that some people who were not in receipt of antidementia drugs before admission had antidementia medication started after discharge. The Cox model allowed a time-varying covariate to be used to account for the time when people were initiated to antidementia treatment after admission within the one-year follow-up period. Analysis was undertaken on an intention-to-treat basis.

### 2.4 Sensitivity analysis

To reduce the effect of treatment selection bias on the primary and secondary outcomes, propensity scores were calculated for each patient defined as the probability to be prescribed antidementia medication based on a patient's demographic characteristics, comorbidities, community prescribing information, and whether the emergency admission was an injury or noninjury admission. Propensity score matching was then used to match each patient in the treatment group that was in receipt of AChEI, or memantine, or both types of drugs to a patient in the reference group. Patients were matched on the logit of propensity score using a caliper of 0.2 standard deviations of the logit of the propensity score [38]. Patients who were initiated to any antidementia drug after discharge were excluded from the cohort before propensity score matching.

A subgroup analysis was conducted separately for the two health-board areas and for patients aged under 85 years to assess the sensitivity of the main results as compared to subgroup results.

Data analysis was carried out using SAS® 9.4 (SAS Institute Inc., Cary, NC, USA).

## 3 Results

### 3.1 Describing the cohort

Between January 2010 and December 2016, there were 27,703 emergency admissions of patients with dementia aged 65 years and over in Tayside and Fife, of which 9142 were incident emergency admissions. Patients with an incident emergency admission were on average 84.2 (95% CI 84.1–84.3) years old, 63.6% (95% CI 62.6–64.6) were women and 23.4% (95% CI 22.6–24.3) were admitted from a care home.

At the time of emergency admission 39.1% (95% CI 38.1–40.1) of patients in the incident cohort were in receipt of antidementia medication. In addition, 5.9% (95% CI 5.4–6.4) had been in receipt of antidementia medication before admission but not on admission, and 4.5% (95% CI 4.1–4.9) were initiated antidementia medication after discharge and within the one-year follow-up time, giving a total of 49.5% of the cohort being exposed to at least one antidementia drug either before or after admission (Fig. 1, Supplementary Table 1). Before or on admission, 31.3% (95% CI 30.4–32.3) of patients were prescribed only AChEIs, 8.7% (95% CI 8.2–9.3) only memantine, and 4.9% (95% CI 4.5–5.4) both classes (Table 1); 46.6% of men were in receipt of antidementia medication before or on admission as compared to 44% of women (difference 2.6%, 95% CI 0.9–5.1, chi-square *P* value = .016). People in receipt of antidementia medication were significantly younger than those receiving no medication (83.2 years vs. 85.0 years on average, difference 1.8 years 95% CI 1.5–2.0, t-test *P* value < .001). Only 39.1% of people in the 85+ age group were in receipt of antidementia medication before or on admission compared to 53.7% in the 70 to 74 age group (Table 2). 36.5% of people admitted from care homes were in receipt of antidementia drug, as compared to 47.5% of people admitted from private home (difference 11.0%, 95% CI 8.6–13.3, *P* value < .001). Antidementia drug prescribing before or on admission was significantly associated with social deprivation with 49.1% versus 43.0% antidementia prescribing rates in least deprived areas SIMD = 5 versus medium class areas of deprivation SIMD = 3 (chi-square *P* value = .003). Among the 10 co-morbidities considered, the antidementia drug prescribing rate was significantly associated with peripheral vascular disease, cerebrovascular disease, and chronic pulmonary disease (all chi-square *P* values < .001), with lower prescribing rates being found in people with these comorbidities. Antidementia drug prescribing before or on admission was also significantly associated with a patient's number of drugs in the 12 weeks before admission (*P* value < .001), and the ACB score in the 12 weeks before admission (*P* value = .015).

**Table 1:** Antidementia medication prescribing rates before or on admission based on patient's characteristics

Patients' characteristics	Any antidementia medication (n = 4110)	AChEIs only (n = 2864)	Memantine only (n = 798)	Both classes (n = 448)
All patients (n = 9142)	45.0 (44.0–46.0)	31.3 (30.4–32.3)	8.7 (8.1–9.3)	4.9 (4.5–5.4)
Sex				
Female (n = 5816)	44.0 (42.7–45.3)	31.7 (30.5–31.7)	7.7 (7.0–8.4)	4.5 (4.0–5.1)
Male (n = 3326)	46.6 (44.9–48.3)	30.6 (29.1–32.2)	10.5 (9.5–11.6)	5.5 (4.8–6.3)
Age group				
65–69 (n = 76)	47.4 (36.6–58.5)	36.8 (26.8–48.0)	4.0 (1.4–11.0)	6.6 (2.9–14.5)
70–74 (n = 607)	53.7 (49.7–57.6)	36.6 (32.9–40.5)	10.2 (8.0–12.9)	6.9 (5.1–9.2)
75–79 (n = 1431)	50.8 (48.2–53.4)	35.4 (33.0–37.9)	8.3 (7.0–9.8)	7.2 (6.0–8.7)
80–84 (n = 2451)	50.3 (48.3–52.3)	34.6 (32.7–36.5)	9.7 (8.6–10.9)	6.0 (5.1–7.0)
85+ (n = 4577)	39.1 (37.7–40.5)	27.5 (26.2–28.8)	8.2 (7.4–9.0)	3.3 (2.8–3.9)
Residential status				
Care home (n = 2143)	36.5 (34.5–38.6)	23.3 (21.6–25.1)	7.8 (6.7–9.0)	5.5 (4.6–6.5)
Living at home (n = 6999)	47.5 (46.3–48.7)	33.8 (32.7–34.9)	9.0 (8.4–9.7)	4.7 (4.2–5.2)
SIMD5∗				
1 most deprived (n = 1275)	44.2 (41.5–46.9)	27.1 (24.7–29.6)	12.7 (11.0–14.6)	4.4 (3.4–5.7)
2 (n = 1571)	45.5 (43.1–48.0)	28.3 (26.1–30.6)	11.4 (9.9–13.1)	5.7 (4.7–7.0)
3 (n = 1869)	43.0 (40.8–45.3)	29.6 (27.6–31.7)	8.5 (7.3–9.9)	4.9 (4.0–6.0)
4 (n = 2664)	43.6 (41.7–45.5)	33.3 (31.5–35.1)	6.3 (5.4–7.3)	4.0 (3.3–4.8)
5 least deprived (n = 1601)	49.1 (46.7–51.5)	36.7 (34.4–39.1)	6.9 (5.8–8.2)	5.5 (4.5–6.7)
Health board				
Fife (n = 4590)	52.2 (50.8–53.6)	29.8 (28.5–31.1)	15.5 (14.5–16.6)	6.9 (6.2–7.7)
Tayside (n = 4592)	37.7 (36.2–39.0)	32.9 (31.6–34.3)	1.9 (1.5–3.4)	2.9 (2.5–3.4)
Comorbidities†				
Myocardial infarction (n = 735)	42.9 (39.4–46.5)	25.4 (22.4–28.7)	13.9 (11.6–16.6)	3.5 (2.4–5.1)
Congestive heart failure (n = 615)	37.9 (34.2–41.8)	22.6 (19.5–26.1)	12.4 (10.0–15.2)	2.9 (1.8–4.5)
Peripheral vascular disease (n = 399)	37.1 (32.5–41.9)	21.1 (17.4–25.4)	12.0 (9.2–15.6)	4.0 (2.5–6.4)
Cerebrovascular disease (n = 1324)	34.4 (31.9–37.0)	22.0 (19.9–24.3)	9.5 (8.0–11.2)	2.9 (2.1–3.9)
Chronic pulmonary disease (n = 1107)	38.9 (36.1–41.8)	23.7 (21.3–26.3)	12.7 (10.9–14.8)	2.6 (1.8–3.7)
Peptic ulcer disease (n = 135)	40.7 (32.8–49.1)	24.4 (17.9–29.6)	15.6 (10.4–22.7)	0.7 (0.1–4.0)
Rheumatic disease (n = 198)	35.4 (29.1–42.3)	23.2 (17.9–29.6)	9.1 (5.8–13.9)	3.0 (1.4–6.4)
Diabetes (n = 1280)	41.4 (38.7–44.1)	27.4 (25.0–29.9)	9.1 (7.6–10.8)	4.8 (3.8–6.1)
Renal disease (n = 1186)	42.6 (39.8–45.4)	26.7 (24.3–29.3)	11.6 (9.9–13.5)	4.3 (3.3–5.6)
Cancer (n = 717)	44.1 (40.5–47.8)	30.1 (26.9–33.6)	9.9 (7.9–12.3)	4.0 (2.8–5.7)
No. of drugs‡				
0 (n = 987)	36.5 (33.6–39.6)	26.8 (24.1–29.6)	4.1 (3.0–5.5)	5.7 (4.4–7.3)
1–5 (n = 5103)	47.1 (45.7–48.6)	33.0 (31.7–34.3)	8.9 (8.1–9.7)	5.3 (4.7–5.9)
6+ (n = 3052)	44.1 (42.3–45.9)	30.1 (28.5–31.8)	10.0 (9.0–11.9)	4.0 (3.4–4.8)
ACB score§				
0 (n = 5834)	44.3 (43.0–45.6)	31.7 (30.5–32.9)	8.3 (7.6–9.0)	4.4 (3.9–5.0)
1–2 (n = 2235)	47.5 (45.4–49.6)	30.7 (28.8–32.6)	10.6 (9.4–11.9)	6.2 (5.3–7.3)
3+ (n = 1073)	43.1 (40.2–46.1)	30.8 (28.1–33.6)	7.4 (6.0–9.1)	4.9 (3.8–6.4)
Emergency type				
Noninjury (n = 7356)	44.6 (43.5–45.7)	30.8 (29.8–31.9)	8.9 (8.3–9.6)	4.9 (4.4–5.4)
Injury (n = 1786)	46.4 (44.1–48.7)	33.4 (31.3–35.6)	8.0 (6.8–9.4)	4.9 (4.0–6.0)

Abbreviations: AChEIs, acetylcholinesterase inhibitors; ACB, anticholinergic burden; ICD-10, International Classification of Diseases.

∗ Scottish Index of Multiple Deprivation divided into five quintiles, 162 values are missing.

† Comorbidity based on ICD-10 codes in SMR01 data set.

‡ Number of drugs prescribed during the 12 weeks before admission.

§ Anticholinergic burden score during the 12 weeks before admission.

**Table 2:** Unadjusted and adjusted HR estimates of Cox model with time-varying covariates for associations between antidementia drug prescribing and mortality with one-year follow-up from admission based on the ITT principle

Patients' characteristics	Model variables	Time period	Unadjusted model HR and 95% CI	Adjusted model HR and 95% CI
Antidementia medication	AChEIs versus no medication		0.65 (0.60–0.70)	0.78 (0.72–0.85)
	Memantine versus no medication		0.74 (0.65–0.83)	0.75 (0.66–0.86)
	Both classes versus no medication		0.68 (0.58–0.79)	0.80 (0.68–0.94)
Sex	Male versus female	Up to 90 days	1.14 (1.04–1.24)	1.15 (1.05–1.27)
		90 days to 1 year	1.40 (1.26–1.54)	1.47 (1.33–1.63)
Age	Per 5 years		1.20 (1.17–1.23)	1.19 (1.16–1.22)
Residence status	Care home versus private home	Up to 30 days	2.46 (2.16–2.79)	2.57 (2.27–2.91)
		30 days to 1 year	1.77 (1.61–1.95)	1.82 (1.66–2.00)
Health board	Fife versus Tayside		1.06 (0.99–1.13)	1.04 (0.97–1.12)
SIMD5∗	1 versus 5 (most vs. least deprived)		1.05 (0.95–1.17)	-
	2 versus 5		1.00 (0.91–1.10)	-
	3 versus 5		1.00 (0.91–1.10)	-
	4 versus 5		0.96 (0.89–1.05)	-
Comorbidities†	Myocardial infarction	Up to 30 days	1.53 (1.27–1.84)	1.30 (1.07–1.57)
(presence vs. absence)		30 days to 1 year	1.05 (0.90–1.22)	0.86 (0.74–1.00)
	Congestive heart failure		1.70 (1.52–1.91)	1.46 (1.29–1.64)
	Peripheral vascular disease		1.83 (1.60–2.09)	1.67 (1.46–1.91)
	Cerebrovascular disease	Up to 30 days	1.69 (1.46–1.95)	1.46 (1.26–1.69)
		30 days to 1 year	1.30 (1.17–1.45)	1.19 (1.06–1.33)
	Chronic pulmonary disease		1.15 (1.04–1.27)	1.11 (1.01–1.23)
	Peptic ulcer disease		1.10 (0.84–1.43)	-
	Rheumatic disease		0.98 (0.78–1.23)	-
	Diabetes		1.01 (0.92–1.11)	-
	Renal disease		1.42 (1.29–1.55)	1.13 (1.08–1.18)
	Cancer—early stage		1.68 (1.49–1.89)	1.66 (1.47–1.87)
	Cancer—metastatic		3.38 (2.84–4.02)	3.77 (3.16–4.49)
No of drugs groups‡	1 to 5		1.08 (0.96–1.21)	1.11 (0.99–1.25)
	6+		1.21 (1.07–1.35)	1.14 (1.00–1.29)
ACB groups§	ACB 1 & 2		1.14 (1.05–1.23)	1.07 (0.98–1.15)
	ACB 3+		1.03 (0.92–1.14)	0.97 (0.87–1.08)
Admission time	Per year	Up to 30 days	0.94 (0.91–0.96)	0.94 (0.91–0.97)
		30 days to 1 year	1.01 (0.99–1.03)	1.01 (0.99–1.03)
Emergency type	Injury versus noninjury	Up to 30 days	0.46 (0.38–0.56)	0.43 (0.36–0.53)
		30 days to 1 year	0.80 (0.71–0.91)	0.84 (0.76–0.93)

NOTE. HR estimates were adjusted for demographic characteristics, comorbidities, community prescribing drugs including ACB, admission time, and type of emergency admission.

Abbreviations: AChEIs, acetylcholinesterase inhibitors; ACB, anticholinergic burden; HR, hazard ratio; ICD-10, International Classification of Diseases; ITT, intention-to-treat.

∗ Scottish Index of Multiple Deprivation divided into five quintiles, 162 values are missing.

† Comorbidity based on ICD-10 codes in SMR01 data set.

‡ Number of drugs prescribed during the 12 weeks before admission.

§ Anticholinergic burden score during the 12 weeks before admission.

### 3.2 Analysis of primary outcome: Time to death in the year after admission

In the year after admission, 38.28% (n = 3500) of patients had died; 32.90% (n = 1352) of patients receiving antidementia medication before or on admission died in the year after admission, compared to 42.7% (n = 2148) of those not receiving antidementia medication before or on admission.

Table 2 shows the associations between antidementia drug exposure and mortality within one year from admission based on intention-to-treat analysis. The results of the Cox model show that after adjustment for demographic characteristics, comorbidities, prescribing, and emergency type, presence of antidementia treatment was significantly associated with a reduction in mortality risk at one year, for both classes of drugs. Specifically, patients in receipt of AChEIs were at a significantly reduced risk of death at one year (HR = 0.78, 95% CI 0.72–0.85), as were patients who were in receipt of memantine alone (HR = 0.75, 95% CI 0.66–0.86) or patients who were receiving both classes (HR = 0.80, 95% CI 0.68–0.94). These results were further confirmed by the secondary analysis, which showed even stronger association between antidementia medication treatment and mortality when only people in receipt of antidementia drug on or after admission were included in the treatment group (Supplementary Materials Table S1).

Male sex, increased age, and residency in a care home were all associated with increased risk of death. The presence of comorbid conditions such as congestive heart failure, peripheral vascular disease, cerebrovascular disease, chronic pulmonary disease, renal disease, and cancer were all significantly associated with an increased risk of death during follow-up. Myocardial infarction was significantly associated with an increase in mortality risk only in the first 30 days from admission. No significant associations were found between peptic ulcer disease, rheumatic disease, or diabetes and increased mortality (HRs in Table 2). Furthermore, an increase in number of drugs prescribed 12 weeks before admission was significantly associated with an increase in mortality risk, while ACB score was not significantly associated with an increased mortality (HRs in Table 2). No significant difference in mortality risks at one year was found between the two health boards. Similar association between patients' demographic, comorbidities, prescribing and emergency type, and mortality was found by the secondary analysis as shown in the Supplementary Table 1.

### 3.3 Sensitivity analysis

The propensity score matching analysis results were consistent with the main results presented previously (Table 3). Indeed, analysis of one-year survival time showed a significant reduction in mortality risk for people in receipt of antidementia drugs on admission, with the highest reduction in those that were in receipt of both drug classes.

**Table 3:** Sensitivity analysis results for associations between antidementia drugs and patients' outcomes after an emergency admission after propensity score matching

Sensitivity analysis	Treatment	ITT analysis (antidementia medication before or on admission)	
		Un-adjusted	Adjusted
Propensity score matching	AChEI only	0.74 (0.67–0.82)	0.73 (0.66–0.81)
	Memantine only	0.78 (0.66–0.92)	0.79 (0.66–0.92)
	Both classes	0.72 (0.54–0.96)	0.69 (0.51–0.93)
Subgroup analysis 84 years old or less	AChEI only	0.67 (0.59–0.75)	0.78 (0.69–0.88)
	Memantine only	0.70 (0.58–0.85)	0.70 (0.58–0.85)
	Both classes	0.75 (0.61–0.92)	0.82 (0.66–1.01)
Subgroup analysis Fife region	AChEI only	0.67 (0.60–0.75)	0.84 (0.74–0.94)
	Memantine only	0.71 (0.62–0.81)	0.77 (0.66–0.87)
	Both classes	0.69 (0.57–0.83)	0.80 (0.66–0.97)
Subgroup analysis Tayside region	AChEI only	0.63 (0.56–0.70)	0.73 (0.66–0.81)
	Memantine only	0.73 (0.51–1.03)	0.80 (0.56–1.13)
	Both classes	0.62 (0.46–0.82)	0.80 (0.59–1.07)

NOTE. Subgroup analysis results for those aged 84 years and younger, and those from region of Fife and Tayside.

Abbreviations: AChEI, acetylcholinesterase inhibitor; ITT, intention-to-treat.

The subgroup analysis for the regions of Fife and Tayside and for those under 85 years were consistent with the analysis of main results (Table 3), with the exception of Tayside, where due to small number of patients under receipt of memantine either alone or combined with AChEI, time to mortality in these treatment groups was not significantly different as compared to those untreated with antidementia medication although the HR was of the same order of magnitude as the full analysis.

## 4 Discussion

Mortality is high in the population with dementia admitted to the acute hospital; over one third of patients died in the year after admission. Forty-five percent (n = 4110) of patients were in receipt of antidementia medication before or on admission, with 31.3% (n = 2864) being prescribed one of the AChEIs, 8.7% (n = 798) memantine, and 4.9% (n = 448) both classes; 32.90% (n = 1352) of these patients died in the year after admission, compared to 42.7% (n = 2148) of those not receiving antidementia medication on admission.

Previous studies have reported associations between antidementia drug use and reduced mortality in the community [18], [21], [22], [23], [39], [40]. There is no previous reporting on associations between antidementia treatment and mortality after admission in this high-risk population of in-patients. People with dementia experience poor outcomes after hospitalization, with higher mortality than people with no dementia [27], [28]. The present study shows for the first time that treatment with antidementia drugs is associated with reduced mortality in the population with dementia. This associated reduction is of the scale that for every 100 patients treated, 10 or fewer would die in the year after admission.

Reduced susceptibility to cardiovascular deaths has been postulated as a possible explanation [41], with one cohort study finding an associated reduction in cardiovascular deaths in people with dementia treated with ACheI [24]. With the ACheI having a direct effect on boosting acetylcholine levels in synapses and hence boosting neurotransmission, modification of this neuropharmacological system is also a candidate when looking at possible explanations to this association with reduced mortality.

Increased anticholinergic burden has been associated with increased risk of mortality in older patients [42]. Associations have been identified between anticholinergic medications and increased dementia risk [43] and between anticholinergic burden and mortality in people with dementia [44]. Our results did not identify an association between anticholinergic burden and mortality risk in patients with dementia. There was however an association with polypharmacy, known to be common in those with dementia [45], and mortality. Frailty has been associated with polypharmacy, although the impact of the association requires further exploration [46]. With the findings that mortality in people with dementia is not associated with disease severity, cognitive function, or functional ability [2], we can postulate that comorbidity and frailty may play a role in determining mortality risk and that it may be via this route that antidementia medication treatment attenuates mortality risk. Of note, expert-derived tools to target polypharmacy in frail older adults could not reach consensus around continuation or stopping antidementia medications, suggesting variation in clinical practice [47].

An important strength of the study is the use of a large, unselected population of people with dementia aged 65 years and over admitted to hospital as a medical emergency, using routine health care national data. To date, this is the largest study reporting on mortality after an emergency admission of people with dementia from two health boards in Scotland. The association between antidementia drug use and mortality is consistent between the two health boards. There are some differences; in particular, we found that memantine is prescribed rarely in Tayside (4.8%) compared to Fife (22.4%). Because of the small number of patients under receipt of memantine (either alone or combined with AChEIs) in Tayside, time to mortality in these treatment group was not significantly different as compared to those free of antidementia medication. However, point estimates of effect size of memantine showed very little difference between the two health boards, suggesting that there are untreated people with dementia that could potentially benefit from the use of this medication.

The main limitation of this study is the potential selection bias in the treatment group, which is characteristic of most observational studies reporting associations between drug use and outcomes. In the case of antidementia drugs, treatment selection bias can be particularly problematic given that up until 2012, NICE guidance in the UK recommended the use of antidementia medication in healthier patients [48]. To address this problem, we conducted a propensity score matching analysis, where patients in receipt of antidementia drug treatment were matched against patients with similar characteristics that were not receiving treatment. The analysis showed results consistent with the main results analysis after adjustment for patients' characteristics, though with perhaps more realistic clinical effects, having reduced confounding. Model estimates were adjusted for patient's demographics, comorbidities, and community prescribing drugs, but no information was available on their functional status or the level of cognitive impairment, although adjusting for these additional factors is debatable due to the risk of overadjustment due to collinearity.

## 5 Conclusions

Mortality in older people admitted with dementia is very high after an emergency hospital admission [28], and we suggest that the population experiencing an acute hospital admission is at increased risk of death compared to those with dementia living in the community. In this study, we show that even in this high-risk population, mortality in people with dementia after a hospital admission is lower in those patients treated with antidementia medication. This finding could be consistent with the suggestion that these classes of medication afford some disease-modifying effect for people with dementia, and postulated mechanisms include reducing cardiovascular deaths and/or attenuating the detrimental effects of comorbidity and frailty. Further research is required to replicate these findings, to determine direct causal relationships between the use of antidementia medication and mortality and to directly elucidate plausible mechanisms for the effects.

Having examined one clinically relevant outcome, mortality, for this study, it does also raise the question as to whether other clinically relevant outcomes (e.g., functional ability or need for long-term care) are modulated in association with dementia treatment in the real-world setting.

**Research in context**

1. Systematic review: The authors searched PubMed for studies that investigated associations between antidementia drugs and mortality in the older population with dementia, with previous research reporting conflicting evidence in this respect. There is no previous report on associations between antidementia treatment and mortality after hospital admission in this population.
2. Interpretation: Mortality in older people admitted with dementia is very high after an emergency hospital admission. However, more than half of the older patients admitted with dementia are not in receipt of any antidementia treatment. Our finding suggests that risk of death is reduced in those patients treated with antidementia medication, either anticholinesterase inhibitors, or memantine or both, which is consistent with some disease-modifying effect of these classes of medication.
3. Future directions: Further research is required to determine direct causal relationships between the use of antidementia medication and mortality in people with dementia.
