# Supplementary material for: Are antidementia drugs associated with reduced mortality after a hospital emergency admission in the population with dementia aged 65 years and older?
Source: Alzheimers Dement (N Y). 2019 Sep 3;5:431–40. doi: 10.1016/j.trci.2019.07.011 (PMC6728828; doi:10.1016/j.trci.2019.07.011)
Supplement: Supplementary Table 1 [file mmc1.docx]

**Supplementary material**

Table S1: Unadjusted and adjusted Cox model HRs for associations between anti-dementia drug prescribing on admission and after admission and mortality with one year follow-up

| Model Variables | | time period | Un-adjusted model HR and 95% CI | Adjusted model HR and 95%CI |
| --- | --- | --- | --- | --- |
| Anti-dementia medication | AChEIs vs no medication |  | 0.56 (0.51-0.61) | 0.67 (0.61-0.73) |
|  | Memantine vs no medication |  | 0.70 (0.62-0.78) | 0.73 (0.64-0.82) |
|  | Both classes vs no medication |  | 0.66 (0.53-0.80) | 0.76 (0.62-0.94) |
| Sex | Male vs Female | up to 90 days | 1.14 (1.04-1.24) | 1.16 (1.05-1.27) |
|  |  | 90 days to 1 year | 1.40 (1.26-1.54) | 1.48 (1.33-1.64) |
| Age | per 5 years |  | 1.20 (1.17-1.23) | 1.19 (1.15-1.22) |
| Residence status | Care home vs private home | up to 30 days | 2.46 (2.16-2.79) | 2.50 (2.21-2.83) |
|  |  | 30 days to 1 year | 1.77 (1.61-1.95) | 1.76 (1.61-1.93) |
| Health board | Fife vs Tayside |  | 1.06 (0.99-1.13) | 1.05 (0.98-1.12) |
| SIMD5 | 1 vs 5 (most vs least deprived) |  | 1.05 (0.95-1.17) | - |
|  | 2 vs 5 |  | 1.00 (0.91-1.10) | - |
|  | 3 vs 5 |  | 1.00 (0.91-1.10) | - |
|  | 4 vs 5 |  | 0.96 (0.89-1.05) | - |
|  | Myocardial infarction | up to 30 days | 1.53 (1.27-1.84) | 1.29 (1.07-1.57) |
| Co-morbidities (presence vs absence) |  | 30 days to 1 year | 1.05 (0.90-1.22) | 0.86 (0.74-1.00) |
|  | Congestive heart failure |  | 1.70 (1.52-1.91) | 1.45 (1.29-1.64) |
|  | Peripheral vascular disease |  | 1.83 (1.60-2.09) | 1.66 (1.45-1.90) |
|  | Cerebrovascular disease | up to 30 days | 1.69 (1.46-1.95) | 1.45 (1.26-1.68) |
|  |  | 30 days to 1 year | 1.30 (1.17-1.45) | 1.18(1.06-1.32) |
|  | Chronic pulmonary disease |  | 1.15 (1.04-1.27) | 1.10 (1.00-1.18) |
|  | Peptic ulcer disease |  | 1.10 (0.84-1.43) | - |
|  | Rheumatic disease |  | 0.98 (0.78-1.23) | - |
|  | Diabetes |  | 1.01 (0.92-1.11) | - |
|  | Renal disease |  | 1.42 (1.29-1.55) | 1.13 (1.08-1.18) |
|  | Cancer - early stage |  | 1.68 (1.49-1.89) | 1.66 (1.47-1.87) |
|  | Cancer - metastatic |  | 3.38 (2.84-4.02) | 3.77 (3.16-4.49) |
| No of drugs groups | 1 to 5 |  | 1.08 (0.96-1.21) | 1.15 (1.02-1.29) |
|  | 6+ |  | 1.21 (1.07-1.35) | 1.18 (1.04-1.34) |
| ACB groups | ACB 1&2 |  | 1.14 (1.05-1.23) | 1.06 (0.98-1.15) |
|  | ACB 3+ |  | 1.03 (0.92-1.14) | 0.96 (0.86-1.07) |
| Admission time | per year | up to 30 days | 0.94 (0.91-0.96) | 0.94 (0.91-0.97) |
|  |  | 30 days to 1 year | 1.01 (0.99-1.03) | 1.01 (0.99-1.03) |
| Emergency type | injury vs non-injury | up to 30 days | 0.46 (0.38-0.56) | 0.43 (0.36-0.53) |
|  |  | 30 days to 1 year | 0.80 (0.71-0.91) | 0.84 (0.76-0.93) |
